# The Work of the Fellowship of Medicine

**Published:** 1920-09-04

**Authors:** 


### POST-GRADUATE TEACHING IN LONDON.

### The Work of the Fellowship of Medicine.

For many years Vienna and Berlin have been the favourite resorts of the graduate student of medicine, and the clinics there have attracted men from all parts of the world, particularly those desiring to study certain specialties. It has taken a great war to bring home to us that we have at our very doors an unsurpassed wealth of clinical material available for post-graduate students, and to show us the imperative need of organising and co-ordinating post-graduate teaching on a cosmopolitan basis. London, not only from its geographical position as the centre between East and West, but from the vastness of the opportunities it affords, should be, and can be, the Mecca of post-graduate students.

Early last year, on the inspiration of Sir Arbuthnot Lane, combined with the business acumen of Sir John MacAlister, a temporary emergency scheme of post-graduate teaching was inaugurated, chiefly with the view of affording facilities to the many medical men from the Colonies and the United States who were expected to visit London on their way home from service overseas.

In the words of our American friends, the scheme "caught on." In little over six months over 600 medical men availed themselves of the facilities provided at the London hospitals under the auspices of the Fellowship of Medicine. It was felt that some such scheme should have a permanent place in London. It was inconceivable that post-graduate students from abroad should be allowed to muddle through on their own, as had been usually the case in the past. With the hearty co-operation and help of the authorities of the various medical schools and special hospitals, the Fellowship of Medicine joined forces with the Post-graduate Association and was formed into a permanent body to organise and co-ordinate the facilities for post-graduate teaching in London.

### General Facilities.

The following hospitals are associated with the Fellowship of Medicine, and on payment of the prescribed fees a ticket can be obtained entitling the holder to attend the general practice of any or all of these institutions:—

Charing Cross Hospital; Guy's Hospital; King's College Hospital Medical School; London Hospital; London (Royal Free Hospital) School of Medicine for Women: Middlesex Hospital; St. Bartholomew's Hospital; St. George's Hospital; St. Mary's Hospital Medical School; St. Thomas's Hospital; University College Hospital Medical School; Westminster Hospital; Cancer Hospital; Central London Ophthalmic Hospital; Central London Throat, Nose and Ear Hospital; Chelsea Hospital for Women; East London Hospital for Children; Great Northern Central Hospital; Hampstead General and North-West London Hospital; Hospital for Diseases of the Throat (Golden Square); Hospital for Sick Children (Great Ormond Street); London Lock Hospital (Dean Street); London School of Tropical Medicine; Margaret Street Hospital for Consumption; National Hospital for the Paralysed and Epileptic; Prince of Wales' General Hospital; Queen's Hospital for Children; Royal Chest Hospital (City Road); Royal Eye Hospital; Royal London Ophthalmic Hospital; St. Mark's Hospital for Diseases of the Rectum; St. Marylebone General Dispensary; St. Peter's Hospital; Seamen's Hospital; Victoria Hospital for Children.

In addition to these general facilities, the Fellowship of Medicine is arranging for special courses of instruction and demonstrations in the various branches of medicine and allied specialties. These courses will last for two or three weeks, and, with the co-operation of the hospital authorities and teachers, it is hoped that it will be possible to arrange that one or more special courses are in progress during the whole of the year.

The *Bulletin* of the Fellowship of Medicine is issued weekly to all members of the Fellowship.

The office of the Fellowship of Medicine is at No. 1 Wimpole Street, London, W. 1 (by kind permission of the Royal Society of Medicine), and the Secretary (Miss M. A. Willis) is in attendance daily from 10 to 5, except on Saturdays.

### Special Courses.

The following special courses and lectures have already been arranged for the autumn:

The course of lectures and demonstrations on "Infant Welfare" will be repeated by Dr. Eric Pritchard at the St. Marylebone General Dispensary, Welbeck Street, W. 1, commencing in September.

A short course of lectures and demonstrations in "Ophthalmology" will be given by Mr. Malcolm L. Hepburn during September.

A course in "Proctology" will be given at St. Mark's Hospital, City Road, E.C. 1, during the weeks Monday, September 20, to Friday, October 1. Lectures will be delivered by Sir C. Gordon Watson, Dr. F. G. Crookshank, Dr. S. D. Wyard, Mr. P. Lockhart Mummery, Mr. H. Graeme Anderson, Mr. Aslett Baldwin, Mr. Lionel E. C. Norbury, and Mr. F. Swinford Edwards.

### Clinical Lectures on Spa Treatment.

If sufficient applications are entered a course of lectures on "Spa Treatment" will be delivered at Bath during the week commencing Monday, September 20. A syllabus, including the following clinical lectures and demonstrations, is now in preparation: Dr. Cave, "Introductory Lecture"; Dr. Munro, "Nature and Properties of Bath Mineral Water"; Dr. Llewellyn, "The Indications for Spa Treatment, and its Limitations"; Dr. Llewellyn, "Gout"; Dr. Thomson, "Fibrositis"; Dr. Leckie, "Demonstration of Pathological Specimens"; Dr. King Martyn; "Massage and Passive Movement in Relation to Spa Treatment," Dr. King Martyn, "Osteo-arthritis"; Dr. Lindsay, "Rheumatoid Arthritis"; Dr. Gordon, "Re-education in Relation to Spa Treatment"; Dr. Llewellyn, "Arthritis Due to Non-specific Infection"; Dr. Waterhouse, "Arthritis Due to Specific Infection"; Dr. Wilson South, "Dietetics in Relation to Spa Treatment"; Dr. Waterhouse, "Sciatica"; Dr. Gordon, "Spa Treatment in Diseases of Nervous System"; Mr. Fraser, "Surgical Potentialities in Relation to Spa Treatment"; Dr. Thomson, "Spa Treatment in Diseases of Cardio-vascular System"; Dr. Lindsay, "Spa Treatment in Diseases of Digestive and Urinary System." The fee for the course is £3 3s., and tickets can be obtained from the Secretary, Fellowship of Medicine, who can also give information as to accommodation, etc.

